# Proteomic Analysis of Golden Sputum Reveals Pulmonary Complement Activation During Acute Chest Syndrome in Children With Sickle Cell Disease

**DOI:** 10.1002/ajh.70182

**Published:** 2025-12-30

**Authors:** Slimane Allali, Nabiha Sbeih, Rachel Rignault‐Bricard, Johanna Bruce, Morgane Le Gall, Claire Heilbronner, Noemie de Cacqueray, Sofia Angyalosy, Melissa Taylor, Joséphine Brice, Mariane de Montalembert, Martina Bevacqua, Emilie‐Fleur Gautier, Olivier Hermine, Thiago Trovati Maciel

**Affiliations:** ^1^ Department of General Pediatrics and Pediatric Infectious Diseases, Sickle Cell Center, Necker‐Enfants Malades Hospital, Assistance Publique–Hôpitaux de Paris (AP‐HP) Université Paris Cité Paris France; ^2^ Laboratory of Cellular and Molecular Mechanisms of Hematological Disorders and Therapeutical Implications Université Paris Cité Imagine Institute, Inserm U1163 Paris France; ^3^ Initiatives IdEx Globule Rouge d'Excellence (InIdex GR‐Ex) Université Paris Cité Paris France; ^4^ Proteom'IC Facility Université Paris Cité, CNRS, INSERM, Institut Cochin Paris France; ^5^ Department of Pediatric Intensive Care, Necker‐Enfants Malades Hospital AP‐HP, Université Paris Cité Paris France; ^6^ Department of Hematology, Necker‐Enfants Malades Hospital, AP‐HP Université Paris Cité Paris France

**Keywords:** acute chest syndrome, child, complement activation, sickle cell disease, sputum

## Abstract

Acute chest syndrome (ACS) is one of the most common severe complications of sickle cell disease (SCD). In recent years, a major role of inflammation and innate immunity has been evidenced, but ACS pathophysiology remains incompletely understood, and therapeutic options are limited. We performed proteomic analysis of induced sputum and tracheal aspirates in eight SCD children during ACS (four intubated and four non‐intubated) and in three during vaso‐occlusive crisis (VOC) without ACS. Proteomic analysis revealed that one of the main canonical pathways involved during ACS was the complement system. To further investigate its implication, we measured the main components of the complement alternative and terminal pathways in sputum and plasma from SCD children during 27 ACS episodes compared with 16 VOC episodes without ACS. A dramatic increase in the median level of C3a, C5a, sC5b‐9, factor B, factor D, properdin, and factor H was observed in the sputum from SCD children during ACS. In the plasma, no significant increase was observed during ACS compared to VOC, except for sC5b‐9, whose median level was twofold higher than during VOC but 16‐fold lower than the sC5b‐9 median level in the sputum during ACS. Also, the C3a/C3 and C5a/C5 ratios were significantly increased in sputum compared to plasma during ACS, reflecting a predominant local pulmonary activation of the complement system compared to systemic activation. Our results reveal the potentially crucial role of complement activation in the lungs during ACS and open new therapeutic perspectives with anti‐complement agents for this severe complication of SCD.

## 1 Introduction

Sickle cell disease (SCD) is a severe inherited hemoglobin disorder, with an increasing incidence of approximately 500 000 newborns affected per year worldwide [1]. It is characterized by chronic anemia, painful vaso‐occlusive events, and a pro‐inflammatory state caused by hemolysis and ischemia/reperfusion injury [2, 3]. Acute chest syndrome (ACS), one of the most frequent life‐threatening complications of SCD, is defined by the association of fever and/or acute respiratory symptoms with a new pulmonary infiltrate on chest imaging [4]. It is the first cause of hospitalization in the intensive care unit (ICU) and a leading cause of premature death of SCD patients [5]. In recent years, growing evidence has highlighted a role of innate immunity and pulmonary inflammation, mediated in particular by interleukin‐6 (IL‐6), in ACS [6, 7]. However, ACS pathophysiology remains unclear, and current therapeutic strategies are mainly supportive, consisting of oxygen and ventilation support, physiotherapy, analgesics, intravenous hydration, empiric antibiotic treatment, preventive anticoagulation, and transfusion when necessary [8]. Besides, golden sputum is considered a pathognomonic sign of ACS, but the origin of the yellowish coloration of sputum, tracheal aspirates, and bronchoalveolar lavage fluids, which is not related to the presence of bilirubin, is still unknown [9]. To try to better understand the complex pathophysiology of ACS, we performed a proteomic analysis of induced sputum and tracheal aspirates in children with SCD during ACS and during vaso‐occlusive crisis (VOC) without ACS. Because proteomic analysis highlighted the complement system as one of the main canonical pathways involved in ACS, we decided to confirm this finding by measuring its main components in sputum and plasma from SCD children during ACS compared with VOC without ACS. Indeed, the complement system is a key player of innate immunity and inflammation, and its involvement in the pathophysiology of SCD has been increasingly recognized [10, 11, 12, 13, 14, 15], especially during VOC [16, 17, 18, 19, 20]. Recent work has shown increased complement activation in the plasma of patients with ACS [21]. Here, we extend these findings by investigating complement locally in the pulmonary compartment through analysis of sputum samples.

## 2 Methods

### 2.1 Study Design

We conducted a prospective exploratory observational study between July 2017 and February 2023 in a French pediatric university hospital SCD reference center. Eligibility criteria were SCD of SS or Sβ^0^ genotype (defining sickle cell anemia), age ≥ 1 year and < 18 years, and hospitalization for VOC or ACS. Exclusion criteria were other diseases leading to increased pulmonary inflammation (e.g., tuberculosis, diagnosis of pneumonia rather than ACS according to the opinion of the investigator, based on the clinical and radiological presentation), use of any other immunomodulatory treatment than hydroxyurea in the last 3 months, and inability to obtain sputum during chest physiotherapy for non‐intubated patients. VOC was defined as an acute episode of pain requiring parenteral opioids, with no other explanation than vaso‐occlusion [22]. ACS was defined as the association of acute respiratory symptoms and/or fever, with a new pulmonary infiltrate on chest x‐ray [4, 8]. In the absence of a consensual definition for ACS severity criteria, those analyzed in our study were as follows: maximal oxygen requirement ≥ 2 L/min, invasive mechanical ventilation, respiratory support (invasive and/or noninvasive) length ≥ 5 days, bilateral or extensive opacities on chest x‐ray, erythropheresis requirement, associated organ (liver, kidney, cardiac, or neurological) dysfunction, hospitalization length in ICU ≥ 7 days, and total hospitalization length ≥ 10 days [23]. Noninvasive ventilation consisted of bilevel positive airway pressure and was performed in all patients not requiring invasive mechanical ventilation, according to our local protocol (all ACS episodes being managed in the ICU in our center) [24]. Sputum was obtained during chest physiotherapy by autogenic drainage [25] or by endotracheal suctioning for intubated patients, performed within the first 72 h of hospitalization for VOC or admission to ICU for ACS. Blood was collected in ethylenediamine tetraacetic acid, and plasma was obtained by centrifugation (10 min, 3500 × *g*, 4°C). Collected sputum and plasma samples were immediately frozen and stored at −80°C. Proteomic analysis of sputum is described in the Supporting Information. All complement measurements were performed at the end of the study, using the ELISA technique (human C3a, HycultBiotech; human C3, Abcam; human complement component C5a, R&D Systems; human C5, Abcam; human C5b‐9, BD Biosciences; human complement factor B, HycultBiotech; human Complement Factor D, R&D Systems; human Properdin, Biolegend; human complement factor H, R&D Systems). Clinical and biological data were obtained from the patient's medical files.

### 2.2 Statistical Analysis

Data are expressed as median [interquartile range (IQR)], or percentage. Differences between groups were assessed using Mann–Whitney, chi‐square, or Fisher's exact tests, as appropriate. Correlation analyses were determined by Spearman's rank test. There was no formal sample size calculation for this exploratory study. The statistical significance threshold was set at a *p* value of 0.05. Statistical analyses were performed using GraphPad Prism version 10 (GraphPad Software, San Diego, USA).

### 2.3 Ethics

Written informed consent was obtained from all children's parents or legal guardians. The study was approved by a medical ethics committee (GR‐Ex/CPP‐DC2016‐2618/CNIL‐MR01).

## 3 Results

### 3.1 Characteristics of Patients

During the study period, 27 ACS and 16 VOC episodes were included in 25 and 16 patients, respectively. Two patients presented two successive ACS episodes. Sputum and plasma were collected after a median delay of 1.5 [1.0–2.0] days after admission. Characteristics of patients and biological parameters at inclusion are summarized in Table 1. A viral infection was identified by nasopharyngeal testing in no VOC episodes and in seven (26%) ACS episodes (SARS‐CoV‐2, coronavirus 229E, and rhinovirus in two episodes each, respiratory syncytial virus and influenza A in one episode each). None of the patients included in the proteomic analysis had a viral infection. Five patients required invasive mechanical ventilation after a median delay of 2 [1.0–2.0] days after admission for ACS. Erythropheresis was performed in seven patients after a median delay of 2 [1.5–2.0] days after admission for ACS.

**TABLE 1:** Main clinical and biological characteristics of patients during VOC and ACS episodes.

	VOC episodes	ACS episodes	*p*
Number	16	27	
Age (years)	12.4 [9.7–16.2]	10.7 [6.8–13.6]	0.11
Female sex, *n* (%)	12 (75)	9 (33)	0.01
SCD genotype			
SS	16 (100)	25 (93)	—
Sβ^0^	0 (0)	2 (7)	—
G6PD deficiency, *n* (%)	1 (6)	8 (30)	0.12
Hydroxyurea, *n* (%)	16 (100)	18 (67)	0.02
MET program, *n* (%)	2 (12)	2 (7)	0.62
Number of VOC since birth	13 [9.5–20]	4 [2–10]	0.01
Number of VOC in the last year	1 [1–3]	1 [0–2]	0.15
Number of ACS since birth	0 [0–1]	1 [0–2]	0.05
Number of ACS in the last year	0 [0–0]	0 [0–0]	0.14
Oxygen requirement, *n* (%)	0 (0)	24 (89)	< 0.001
Oxygen ≥ 2 L/min, *n* (%)	—	19 (70)	—
Invasive/noninvasive ventilation, *n* (%)	—	5 (19)/22 (81)	—
Ventilation length (days)	—	5 [3–9]	—
Bilateral/extensive opacities (chest x‐ray)	—	21 (78)	—
Acute organ dysfunction^a^	0 (0)	4 (15)	0.28
Erythropheresis	0 (0)	7 (26)	0.03
Hospitalization length in ICU (days)	0 [0–0]	8 [5.5–10.5]	< 0.001
Total hospitalization length (days)	3 [2–5]	11 [10–14.5]	< 0.001
Hb level (g/dL)	9.2 [8.4–10.1]	7.7 [6.5–8.8]	0.001
Reticulocyte count (G/L)	220 [161–328]	346 [230–423]	0.04
Leukocyte count (G/L)	9.7 [8.4–11.4]	18.7 [12.5–26.6]^b^	< 0.001
Absolute neutrophil count (G/L)	5.5 [2.9–6.1]	15.3 [9.0–23.1]^b^	< 0.001
Monocyte count (G/L)	1.1 [0.8–1.4]^b^	3.3 [2.4–3.6]^b^	0.003
Platelets (G/L)	375 [195–475]	241 [151–328]	0.07
CRP (mg/L)	4 [3–7]^b^	147 [106–196]	< 0.001
AST (U/L)	50 [40–61]^c^	46 [35–69]^d^	0.82
Unconjugated bilirubin (μmol/L)	20 [15–33]^c^	27 [20–55]	0.09
LDH (U/L)	501 [365–648]^c^	630 [468–854]^d^	0.23

*Note:* Data are expressed as median [interquartile range] or percentage.

Abbreviations: ACS, acute chest syndrome; AST, aspartate aminotransferase; CRP, C‐reactive protein; G6PD, glucose‐6‐phosphate dehydrogenase; Hb, hemoglobin; ICU, intensive care unit; LDH, lactate dehydrogenase; MET, manual exchange transfusion; SCD, sickle cell disease; VOC, vaso‐occlusive crisis.

^a^ Liver, kidney, cardiac, or neurological dysfunction.

^b^ One missing data.

^c^ Six missing data.

^d^ Three missing data.

### 3.2 Proteomic Analysis of Sputum Reveals That the Complement System Is One of the Main Canonical Pathways Involved During ACS

**FIGURE 1:**
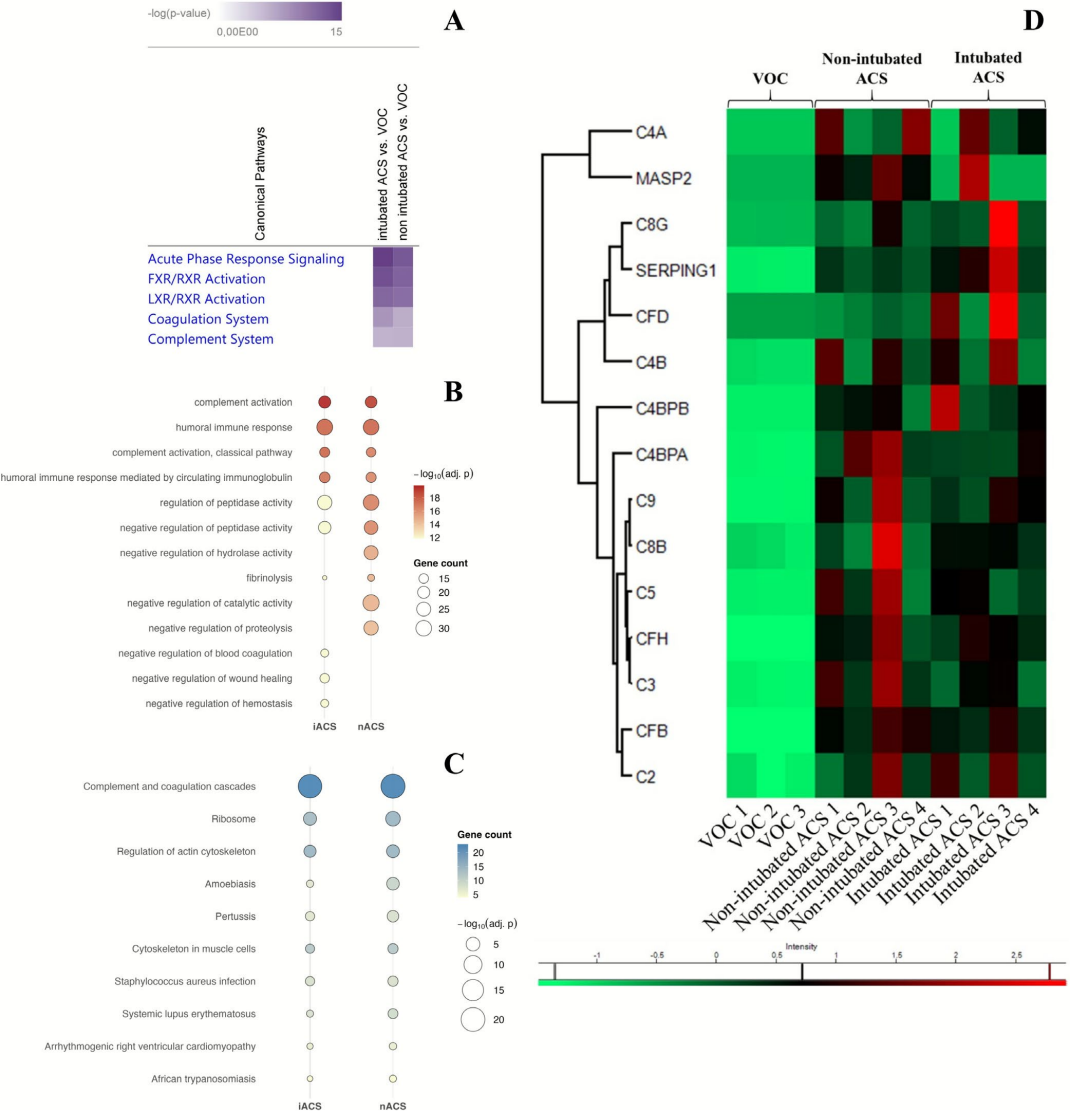
(A–C) Heatmaps generated by ingenuity pathways analysis (IPA) (A), Gene Ontology (GO) (B), and KEGG pathway enrichment analysis (C) showing the most enriched pathways in both comparisons of sputum and tracheal aspirates from SCD patients during VOC (*n* = 3), intubated ACS (iACS, *n* = 4), and non‐intubated ACS (nACS, *n* = 4). Color gradient reflects the enrichment's significance (−log‐*p* value). (D) Heatmaps of proteins involved in the complement system, highlighted by proteomic analysis of sputum and tracheal aspirates from SCD patients during VOC (*n* = 3), non‐intubated ACS (*n* = 4), and intubated ACS (*n* = 4). Heatmaps were generated by Perseus. Hierarchical clustering was performed on *z*‐scored label‐free quantification (LFQ) intensities using Pearson correlation with average linkage. ACS, acute chest syndrome; VOC, vaso‐occlusive crisis. [Color figure can be viewed at wileyonlinelibrary.com]

To try to decipher the complex pathophysiology of ACS, we first performed proteomic analysis of induced sputum and tracheal aspirates in eight SCD children during ACS (four intubated and four non‐intubated) and in three SCD children during VOC without ACS (Table S1). As a quality control step, we compared the 2896 quantified proteins in these sputum samples to reference proteomes of various biofluids using Fisher's exact test (Figure S1). The samples showed significant enrichment for sputum‐related terms and closely matched published sputum proteomes [26], while displaying lower enrichment for plasma and saliva proteomes, thereby confirming the suitability of the material for downstream analysis. Overrepresentation analysis using QIAGEN IPA (QIAGEN Inc., https://digitalinsights.qiagen.com/IPA) revealed that one of the main canonical pathways involved during ACS compared with VOC was the complement system (Figure 1A). To further characterize the biological processes underlying ACS in children with SCD, we performed term enrichment analyses (Gene Ontology [GO] and KEGG) on proteins differentially expressed in sputum from ACS compared with VOC and proteins only identified in one of the compared groups. These analyses consistently highlighted an enrichment of proteins related to innate immunity and inflammation, with a predominance of complement‐associated pathways. In the GO analysis (Figure 1B), top‐enriched biological processes in both intubated and non‐intubated ACS groups included “complement activation” (GO: 0030449), “complement activation, classical pathway” (GO: 0006958), and “humoral immune response” (GO: 0006959). KEGG analysis confirmed this result (Figure 1C), with “Complement and coagulation cascades” (hsa04610) emerging as the top‐enriched pathway. These terms ranked among the most significant (FDR < 0.05), reflecting the overrepresentation of complement components such as C3, C4, C5, factor B, factor H, and properdin in ACS sputum (Figure 1D and Figure S2). Both alternative and terminal pathway proteins were upregulated, pointing to a coordinated activation of complement during ACS. When comparing intubated and non‐intubated patients, the degree of enrichment was greater in the intubated group, suggesting that complement activation is more pronounced in severe ACS requiring mechanical ventilation. Other main canonical pathways identified by IPA software in the proteomic dataset were acute phase response signaling, FXR/RXR and LXR/RXR activation, and the coagulation system (Figure 1A and Figure S3).

### 3.3 Alternative and Terminal Pathways of Complement Are Activated in the Lungs of SCD Patients During ACS

**FIGURE 2:**
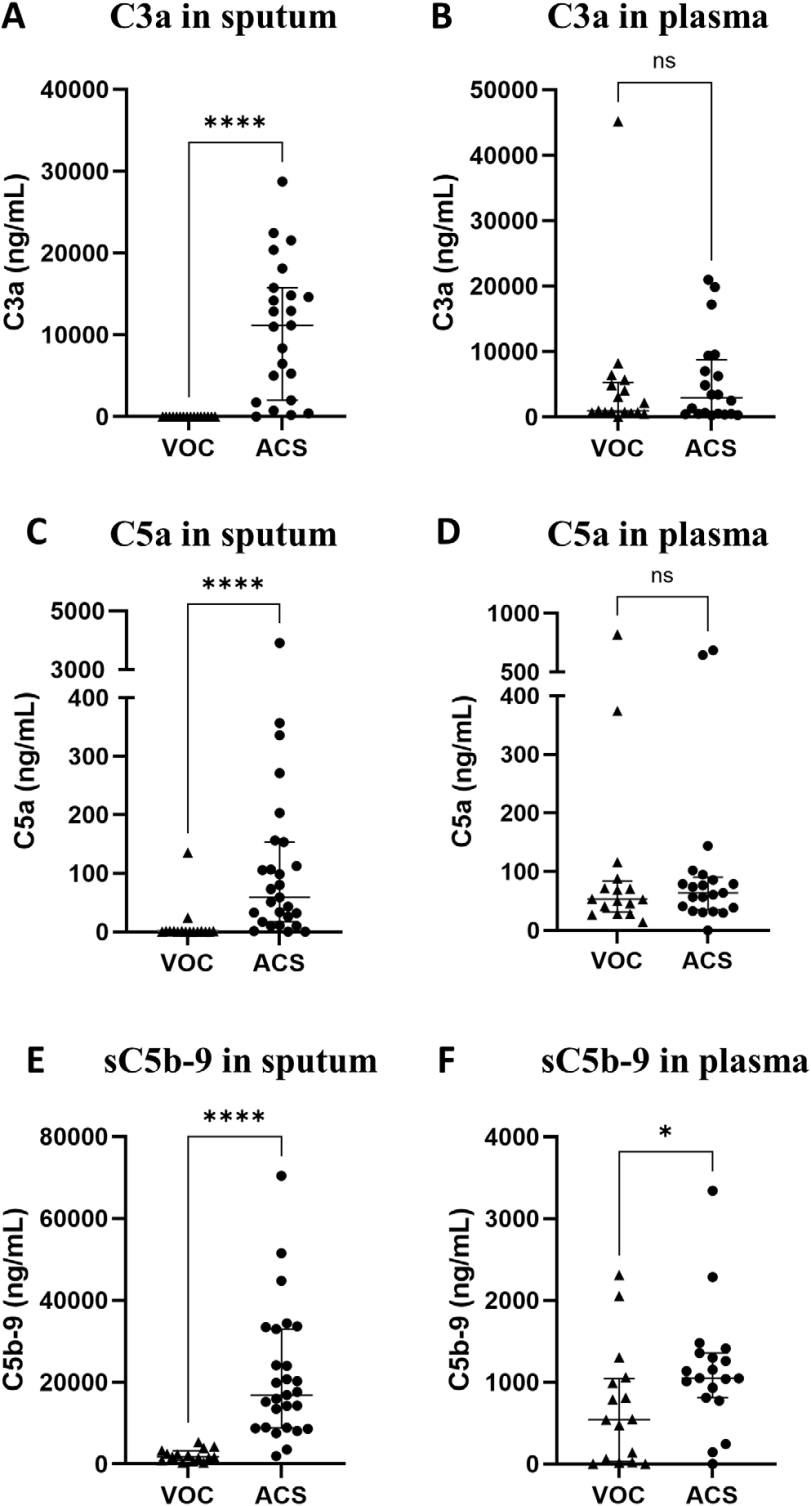
Comparison of the levels of the complement activation products C3a (A, B), C5a (C, D), and sC5b‐9 (E, F) in sputum or tracheal aspirates (A, C, E) and in plasma (B, D, F) from SCD patients, between ACS (*n* = 27 episodes) and VOC without ACS (*n* = 16 episodes). ACS, acute chest syndrome; VOC, vaso‐occlusive crisis. ****p < 0.0001; *p < 0.05 by Mann–Whitney test.

**FIGURE 3:**
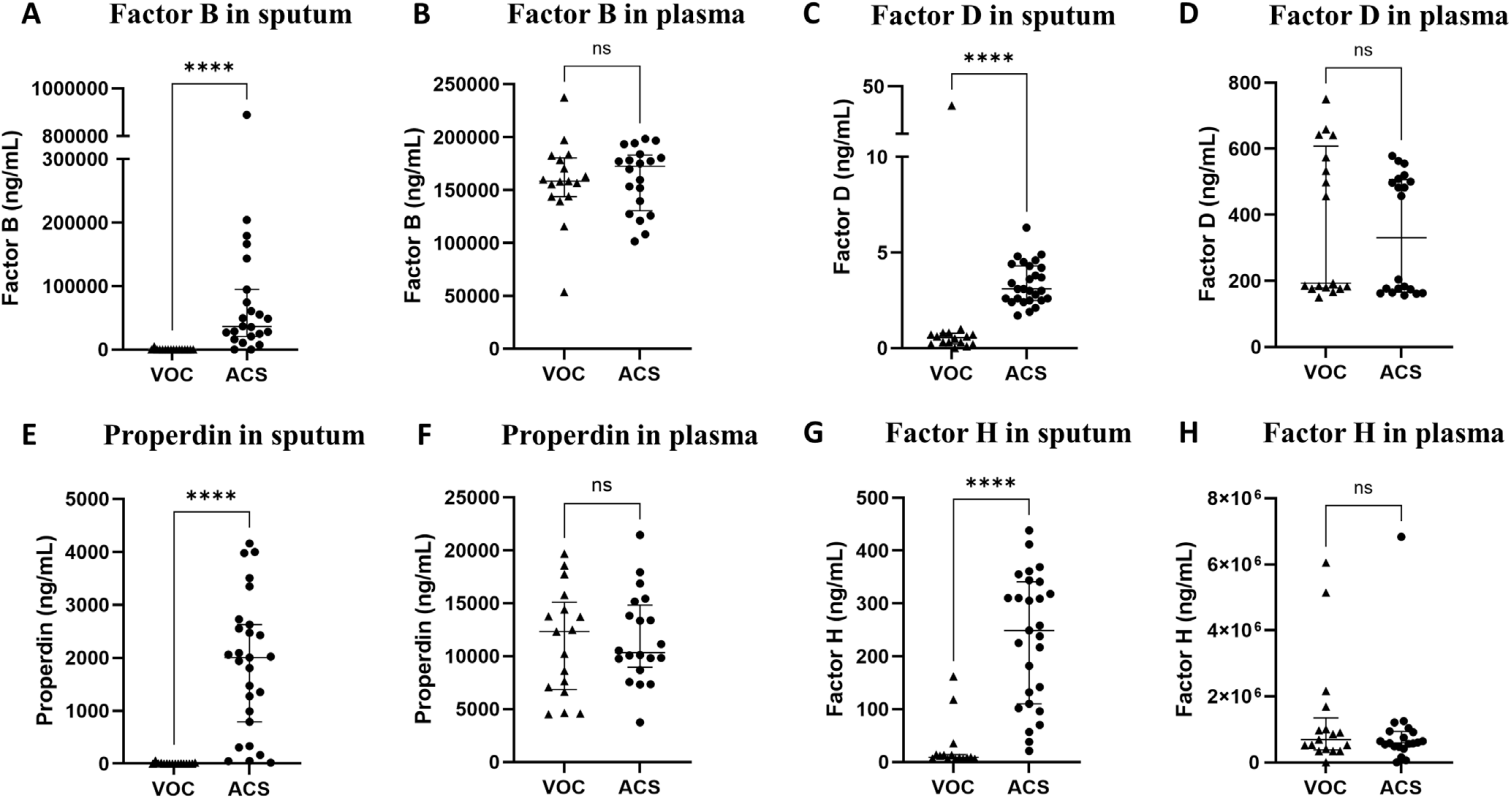
Comparison of the levels of the alternative pathway actors factor B (A, B), factor D (C, D), properdin (E, F), and factor H (G, H) in sputum or tracheal aspirates (A, C, E, G) and in plasma (B, D, F, H) from SCD patients, between ACS (*n* = 27 episodes) and VOC without ACS (*n* = 16 episodes). ACS, acute chest syndrome; VOC, vaso‐occlusive crisis. ****p < 0.0001 by Mann–Whitney test.

**FIGURE 4:**
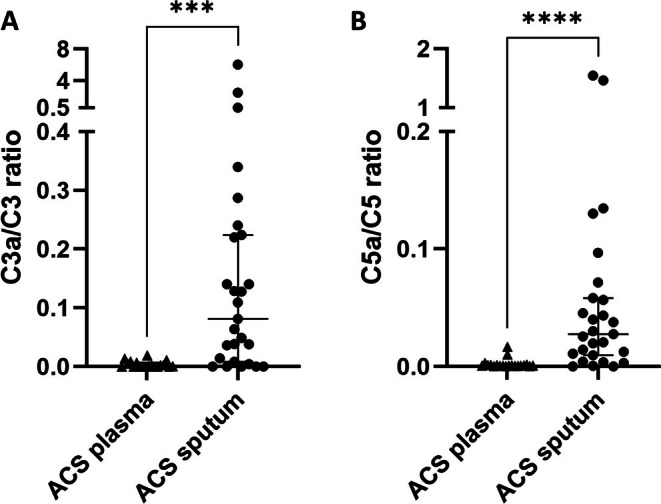
C3a/C3 ratio (A) and C5a/C5 ratio (B) in plasma and in sputum or tracheal aspirates from SCD patients during ACS (*n* = 27 episodes). ACS, acute chest syndrome. ****p < 0.0001; ***p < 0.001 by Mann–Whitney test.

Because growing evidence has highlighted a role of the complement system in SCD that had not been extensively explored during ACS, we then measured its main components in the sputum and plasma of the 41 children enrolled in our study, including the 11 children in whom proteomic analysis had been carried out. The levels of C3a, C5a, sC5b‐9, factor B, factor D, properdin, and factor H in sputum and plasma were compared between the 27 ACS episodes and the 16 VOC episodes without ACS (Figures 2 and 3). In the sputum during ACS compared with VOC, a dramatic increase in the median level of C3a (11 137 [3496–15 292] vs. 0 [0–0] ng/mL, *p* < 0.0001), C5a (59 [21.5–132.5] vs. 0.5 [0–1] ng/mL, *p* < 0.0001), sC5b‐9 (16 872 [8912–28 525] vs. 1745 [1172–2927] ng/mL, *p* < 0.0001), factor B (36 718 [22 768–84 375] vs. 0 [0–0] ng/mL, *p* < 0.0001), factor D (3.1 [2.5–4.2] vs. 0.5 [0.3–0.7] ng/mL, *p* < 0.0001), properdin (2007 [889–2591] vs. 4 [0–6] ng/mL, *p* < 0.0001), and factor H (249 [121–329.5] ng/mL vs. 9 [6.5–13.5] ng/mL, *p* < 0.0001), was observed. In the plasma, no significant increase was observed during ACS compared to VOC, except for sC5b‐9, whose median level was twofold higher than during VOC (1049 [872.5–1326] vs. 544 [50–1006] ng/mL, *p* = 0.03) but 16‐fold lower than sC5b‐9 median level in the sputum during ACS (1049 [872.5–1326] vs. 16 872 [8912–28 525] ng/mL, *p* < 0.0001). Also, the C3a/C3 ratio was significantly higher in sputum than in plasma during ACS (81.4 [11.2–222] × 10^−3^ vs. 1.8 [0.3–5.9] × 10^−3^, *p* = 0.0005) and the C5a/C5 ratio was also increased (27.7 [10.2–57.4] × 10^−3^ vs. 0.8 [0.6–1.4] × 10^−3^, *p* < 0.0001), reflecting a predominant local pulmonary activation of the complement system compared to systemic activation (Figure 4). The levels of C3 and C5 in sputum and plasma during ACS and VOC are reported in Figure S4.

### 3.4 The Level of Soluble C5b‐9 in Sputum Is Negatively Correlated With the Platelet Count and Positively Correlated With CRP Level

No significant correlations were observed between any clinical or biological parameter and the levels of complement components in sputum, except for sC5b‐9 level, which was negatively correlated with the platelet count (*r* = −0.39, *p* = 0.04) and positively correlated with CRP level (*r* = 0.42, *p* = 0.03). Also, factor H level was positively correlated with AST (*r* = 0.49, *p* = 0.01) and LDH (*r* = 0.49, *p* = 0.02) levels. Patients with bilateral or extensive opacities on chest x‐ray had a higher median level of C5a (80 [33–156] vs. 13.5 [3.2–36.5] ng/mL, *p* = 0.04) and C5a/C5 (37.8 [14.2–71.6] × 10^−3^ vs. 7.5 [1.4–18.2] × 10^−3^, *p* = 0.04) in sputum than patients without (Figure S5). No difference in the levels of complement components was observed between patients with or without hydroxyurea.

## 4 Discussion

Here, we report a novel involvement of the complement system in the pathophysiology of ACS, with increased levels of C3a, C5a, sC5b‐9, factor B, factor D, properdin, and factor H in the sputum and tracheal aspirates of SCD children during ACS compared with VOC without ACS. By contrast, no significant increase was observed in plasma during ACS compared to VOC, except for sC5b‐9 plasma level, which was twofold higher than during VOC but 16‐fold lower than sC5b‐9 sputum level during ACS. This, together with increased C3a/C3 and C5a/C5 ratios in sputum compared to plasma, suggests local activation of the complement system in the lungs during ACS.

In the last years, growing evidence has highlighted a role of the complement system in SCD. Increased levels of several components of the alternative and terminal pathways, including Bb, C3a, C3b‐properdin complexes, C5a, and sC5b‐9, have been reported in the plasma from SCD patients compared to controls, both at steady state and during VOC [10, 12, 15, 16, 17, 18, 27, 28, 29]. Besides, deposition of complement products has been described in several organs from patients with SCD, including C3 and C9 in the kidney and C5b‐9 in the skin [13, 19]. Similarly, in transgenic sickle mice, increased C3 and C5b‐9 deposits have been evidenced in the kidney, but also in the liver and the lung after a challenge with hemoglobin or hypoxia‐reoxygenation [13, 20]. Besides, transcriptomic analysis of these three organs in HbSS Townes mice revealed upregulation of complement genes compared to HbAA mice [30]. Suspected mechanisms underlying complement activation in SCD involve the sickle red blood cells (RBCs) themselves, on the surface of which C3d was found to be increased at steady state and even more during VOC, with a possible contribution of increased phosphatidylethanolamine and phosphatidylserine exposure on their surface compared to normal RBCs [16, 18, 19]. Intravascular hemolysis may also play an important role through the release of free heme, which is a well‐known activator of the complement alternative pathway. Indeed, exposure of normal human serum to heme results in increased levels of C3a, C5a, and sC5b‐9 [31]. In addition, heme promotes C3 deposition at the surface of normal RBCs as well as C3b and C5b‐9 deposition on cultured human endothelial cells, thus mimicking the effect observed after exposure to serum from patients with SCD, an effect diminished by the presence of the heme scavenger hemopexin [15, 32]. In transgenic sickle mice, heme was shown to trigger vaso‐occlusion via TLR4 signaling on endothelial cells, leading to Weibel–Palade body degranulation with the release of von Willebrand factor (vWF) and the adhesion molecule P‐selectin [33]. Interestingly, the same effect was observed after infusion of purified C5a in HbSS mice, which resulted in increased vWF and P‐selectin release, together with increased TLR4 expression [14]. In this model, microvascular stasis was prevented by both anti‐C5a receptor and anti‐P‐selectin antibodies. Terminal pathway component C5b‐9 may also trigger secretion of vWF and P‐selectin expression on endothelial cells [29]. The close interconnection between complement activation, intravascular hemolysis, and endothelial cell activation that promotes adhesion is consistent with the hypothesis of a role of the complement system in ACS pathophysiology. Indeed, heme infusion is the main model of ACS induction in sickle cell mice, and this effect was found to be mediated by P‐selectin [34, 35]. In SCD patients, high plasma‐free heme level was associated with increased incidence of ACS [36]. During VOC, ACS was found more likely to develop in case of high reticulocyte counts [37], and a negative correlation was observed between hemoglobin level and sputum IL‐6 level [38], which supports the hypothesis of a role for hemolysis in ACS pathophysiology. In our study, a positive correlation was observed between two hemolysis markers, AST and LDH, and factor H, which is recognized as the main regulator of the alternative pathway of the complement system [39]. No correlation was observed with the other complement activation markers, notably sC5b‐9, but it might be due to a lack of statistical power in the context of a small sample size. On the other hand, sputum sC5b‐9 level was found to correlate positively with CRP level and negatively with platelet count, which is worth noting because increased CRP level and decreased platelet count are known risk factors and markers of severity of ACS [4, 23, 38, 40]. At diagnosis of ACS, a low platelet count < 200 G/L was found to be associated with respiratory failure and prolonged hospitalization [4]. This could reflect consumption or recruitment of platelets to the lungs. Pulmonary platelet thrombi have been described in 30% of autopsies from SCD patients who died from ACS, together with increased endothelial deposition of vWF, which may contribute to platelet adhesion and occlusion in the microvasculature [41]. In mice, neutrophil–platelet aggregates in lung arterioles have been shown to promote lung vaso‐occlusion during ACS, with a preventive effect of P‐selectin inhibition [42]. Also, sterile inflammation may induce the formation of neutrophil extracellular traps that travel intravascularly from liver to lung, where they promote neutrophil–platelet aggregation and acute lung injury [7]. Interactions between platelets and the complement system have been well described, and activated platelets could contribute to the activation of the alternative pathway in SCD [43]. Complement activation may promote acute inflammation, which is a hallmark of ACS [6], as C3a and C5a are recognized as potential pro‐inflammatory mediators [44]. On the other hand, inflammation might also contribute to complement activation in the lungs, because IL‐6, whose levels are increased in sputum from SCD patients during ACS [6, 23], is able to stimulate the synthesis of C3 and factor B [45].

Interestingly, the other main canonical pathways highlighted by the proteomic analysis of sputum during ACS were acute phase response signaling, the coagulation system, whose interaction with the complement system is well established [46], and activation of FXR/RXR and LXR/RXR, known to modulate lipid homeostasis. The involvement of these two last pathways might reflect the potential contribution of fat embolism secondary to bone marrow necrosis in ACS [47], with the generation of fatty acids by secretory phospholipase A2 (sPLA2) that promote lung injury [8, 48]. It might also reflect the intricate interplay between lipid homeostasis and the complement system [49].

Our findings on the involvement of the complement system in ACS pathophysiology are consistent with very recently reported data on the role of complement activation in inducing ACS in a mouse model of SCD, with a preventive effect of complement inhibition on heme‐induced acute lung injury [21]. The authors also found that in SCD patients, plasma C3a, C5a, and sC5b‐9 levels were increased during ACS compared to baseline. However, the levels of these alternative and terminal complement pathway components were not analyzed in sputum or tracheal aspirates, and plasma levels could also reflect systemic complement activation during VOC, which is frequently associated with ACS.

Here, we identify the complement system as a key biological signature within the pulmonary milieu of children with ACS. The concurrent presence of both effector and regulatory proteins suggests a finely tuned but ultimately insufficient balance between activation and control occurring locally in the lung. This observation reinforces the findings from experimental SCD models where alternative pathway activation drives vascular injury and inflammation. By extending these insights to human ACS, our study underscores the importance of exploring therapeutic approaches that specifically target complement activation in the pulmonary compartment. The anti‐C5 monoclonal antibody, eculizumab, is increasingly used for delayed hemolytic transfusion reactions (DHTRs) [50, 51], and respiratory improvement was reported in a patient with severe ACS who received eculizumab for DHTR [50]. Also, two ongoing clinical trials on the novel self‐injectable anti‐C5 monoclonal antibody crovalimab, for the prevention (NCT05075824) and the treatment (NCT04912869) of vaso‐occlusive episodes, are planned to investigate ACS occurrence as a secondary outcome.

There are several limitations to our study. First, patients who experienced an episode of ACS were not the same as those who experienced an episode of VOC. Thus, the proportion of patients treated with hydroxyurea was lower in the ACS group than in the VOC group, which could have influenced our results if we assume a protective effect of hydroxyurea on complement activation. However, no difference in the levels of complement components was observed between patients treated with and those not treated with hydroxyurea. Second, a selection bias might exist due to the marked severity of the patients followed in our SCD reference center, which may have resulted in particularly high levels of complement components during ACS. Multicenter studies with a better representation of the less severe forms of ACS are therefore warranted. Third, the limited number of patients included in our study may have resulted in insufficient statistical power, and larger studies will need to be considered in the future. Fourth, a measurement bias could exist as the ACS definition we used, although consensual, lacks specificity. Thus, pneumonias might have been misclassified as ACS. Also, sputum was collected up to 3 days after admission, and earlier collection within the first hours of ACS diagnosis would be necessary to determine whether complement activation could be predictive of ACS severity.

In conclusion, the complement system appears as a new promising actor of ACS pathophysiology with predominant local pulmonary activation. Double‐blind, randomized, placebo‐controlled clinical trials are needed to assess the efficacy and safety of anti‐complement agents for treating this severe and frequent complication of SCD.

## Author Contributions

S.A., N.S., R.R., and T.T.M. collected, analyzed, and interpreted data and wrote the manuscript. J.B., M.L., C.H., J.B., M.M., C.H., N.C., S.A., M.T., J.B., M.M., E.G., and O.H. helped in the discussions, assisted in writing, and finalized the manuscript.

## Funding

This work was supported by state funding from the Agence Nationale de la Recherche under the Investissements d'avenir Program (ANR‐10‐IAHU‐01), DIM Thérapie Génique Paris Ile‐de‐France Région, IBiSA, and the France 2030 Program Through the Idex Université Paris Cité, InIdex GR‐Ex (ANR‐18‐IDEX‐0001).

## Conflicts of Interest

The authors declare no conflicts of interest.

## Supporting information


**Data S1:** Supporting Information.

## Data Availability

The data that support the findings of this study are available from the corresponding author upon reasonable request.
