# Supplementary material for: Proteomic Analysis of Golden Sputum Reveals Pulmonary Complement Activation During Acute Chest Syndrome in Children With Sickle Cell Disease
Source: Am J Hematol. 2025 Dec 30;101(4):678–86. doi: 10.1002/ajh.70182 (PMC12994127; doi:10.1002/ajh.70182)
Supplement: Supplementary file 1 — Data S1: Supporting Information. [file AJH-101-678-s001.docx]

**Proteomic analysis of golden sputum reveals pulmonary complement activation during acute chest syndrome in children with sickle cell disease**

**SUPPLEMENTARY METHODS**

*Proteomic analysis of sputum*

Sputum sample were solubilized in lysis buffer (2% SDS, 200mM Tris-HCl, pH 8.5, 10 mM TCEP, 50 mM CAA) and boiled 5 minutes at 95°C. 30µg of each sample were digested using 1µg trypsin (Promega) and S-Trap Micro Spin Column was used according to the manufacturer’s protocol (Protifi, Farmingdale, NY, USA). Peptides were dried in a SpeedVac, resuspended in 500 µL of Buffer A (10 mM ammonium formate, pH 10 in mQ H2O) and sonicated for five minutes before being loaded onto an AKTA chromatography system equipped with a Zorbax Extend-C18 column (Agilent, Santa Clara, USA) for Reverse-Phase High pH (RP-HpH) peptide fractionation. Peptides bound to the hydrophobic C18 phase were eluted with Buffer B (10 mM ammonium formate, pH 10, in 80% acetonitrile). Next, 250 µL fractions were collected at a flow rate of 0.250 mL/min during 1h24min (total vol. = 21 mL) on three different gradients (Buffer A and B mixed) with an initial (from 0 to 2 mL) flow rate at 6.25% of buffer B (93.75% of Buffer A): (1) 6.25–44% of Buffer B (from 2 mL to 14.5 mL), (2) 44–75% of Buffer B (from 14.5 mL to 17 mL), and (3) 75–100% of Buffer B (from 17 mL to 21 mL). The 84 harvested fractions were pooled into nine final fractions based on a concatenation plan to equalize the amount of peptides in each fraction. The nine fractions were subsequently dried using a SpeedVac.

*Liquid Chromatography-coupled Mass spectrometry analysis (nLC-MS/MS)*

nLC-MS/MS analyses were performed on a Dionex U3000 HPLC nanoflow chromatographic system (Thermo Fischer Scientific) coupled to a TIMS-TOF Pro mass spectrometer (Bruker Daltonik GmbH, Bremen, Germany). After drying, the nine fractions of peptides were solubilized in 10μL of 0.1% TFA containing 10% acetonitrile (ACN). five μL were loaded, concentrated and washed for 3min on a C_18_ reverse phase column (5μm particle size, 100 Å pore size, 300 μm inner diameter, 0.5 cm length, from Thermo Fisher Scientific). Peptides were separated on an Aurora C18 reverse phase resin (1.6 μm particle size, 100Å pore size, 75μm inner diameter, 25cm length mounted to the Captive nanoSpray Ionisation module, from IonOpticks, Middle Camberwell Australia) with a 60 minutes overall run-time gradient from 99% of solvent A containing 0.1% formic acid in milliQ-grade H2O to 40% of solvent B containing 80% acetonitrile, 0.085% formic acid in mQH2O with a flow rate of 400 nL/min. The mass spectrometer acquired data throughout the elution process and operated in DDA PASEF mode with a 1.17 second/cycle. Ion accumulation and ramp time in the dual TIMS analyzer were set to 100 ms each and the ion mobility range was set from 1/K0 = 0.6 Vs cm-2 to 1.6 Vs cm-2. Precursor ions for MS/MS analysis were isolated in positive polarity with PASEF in the 100-1.700 m/z range. The cycle duty time was set to 100%. Precursors for MS/MS were picked from an intensity threshold of 1000 and re-fragmented and summed until reaching a ‘target value’ of 20.000 a.u.

*Protein identifications and quantifications*

The mass spectrometry data were analyzed using Maxquant version 2.0.3.0.^1^ The database used for in silico generation of spectral library was a concatenation of Human sequences from the Swissprot database (release 2022-09) and a list of contaminant sequences from Maxquant and from the cRAP (common Repository of Adventitious Proteins). The enzyme specificity was trypsin. The precursor and fragment mass tolerances were set to 20ppm. Acetylation of the protein N terminus and oxidation of methionines was set as variable modifications while carbamidomethylation of cysteines was set as fixe modification. Precursor false discovery rate (FDR) was kept below 1%. The “match between runs” (MBR) and the normalization option was allowed. The results files of Maxquant were analysed with Perseus version 1.6.15.0.^2^ Contaminants and reverse proteins were eliminated. The data were transformed to Log 2 and only proteins with 3 valid values in at least one group were kept. A two-tailed independent-samples t-test was applied to identified differential proteins. Were considered as proteins of interest between two groups, proteins showing both Benjamini-Hochberg adjusted p-value below 0.05 and absolute fold change greater than 1.5.

*Functional analysis*

Functional analyses were generated through the use of Ingenuity Pathways Analysis (IPA, QIAGEN Inc., [https://www.qiagenbioinformatics.com/products/ingenuitypathway-analysis, version](https://www.qiagenbioinformatics.com/products/ingenuitypathway-analysis,%20version) 1348237) for each list of differential proteins. Over-represented canonical pathways and terms related to biofluids were highlighted using a right-tailed Fisher’s Exact Test that calculates a statistical significance of overlap of modulated proteins with annotation gene sets. Significant proteins were also subjected to functional enrichment analysis for Gene Ontology (GO) biological processes and Kyoto Encyclopedia of Genes and Genomes (KEGG) pathways. Protein identifiers were converted to Entrez gene IDs using the *bitr* function from the clusterProfiler package. Over-representation analyses were performed with FDR correction, and enrichment results were visualized as bubble plots showing –log10 adjusted p-values in R (ggplot2 package). To explore co-regulation patterns, standardized log2-transformed expression values were analyzed by hierarchical clustering using Ward’s method. Clusters were defined by dynamic tree cutting, and functional coherence of each cluster was evaluated with g:Profiler, testing enrichment against GO and KEGG categories. Hierarchical clustering was generated using Perseus (v.1.6.15.0). Label-Free Quantification (LFQ) intensities were z-scored before performing Pearson correlation with average linkage.

**SUPPLEMENTARY TABLES**

**Table S1.** Main clinical and biological characteristics of patients included in the proteomic analysis

|  | *VOC episodes* | *Non-intubated*  *ACS episodes* | *Intubated*  *ACS episodes* |
| --- | --- | --- | --- |
| *Number* | 3 | 4 | 4 |
| *Age (years)* | 11.9 [9.9-17.2] | 10.3 [9.2-16.8] | 4.9 [2.8-13.2] |
| *Female sex, n (%)* | 2 (67) | 1 (25) | 1 (25) |
| *SCD genotype:* |  |  |  |
| - *SS* | 3 (100) | 4 (100) | 4 (100) |
| - *Sβ^0^* | 0 (0) | 0 (0) | 0 (0) |
| *G6PD deficiency, n (%)* | 0 (0) | 0 (0) | 1 (25) |
| *Hydroxyurea, n (%)* | 3 (100) | 1 (25) | 1 (25) |
| *MET program, n (%)* | 1 (33) | 1 (25) | 0 (0) |
| *Number of VOC since birth* | 13 [9-20] | 3.5 [3-5] | 2.5 [0-19] |
| *Number of VOC in the last year* | 1 [0-2] | 0.5 [0-1] | 0.5 [0-4] |
| *Number of ACS since birth* | 2 [0-3] | 0 [0-3] | 1 [0-2] |
| *Number of ACS in the last year* | 0 [0-0] | 0 [0-1] | 0 [0-0] |
| *Oxygen requirement, n (%)* | 0 (0) | 3 (75) | 4 (100) |
| *Oxygen ≥ 2 L/min, n (%)* | - | 4 (100) | 4 (100) |
| *Invasive/non-invasive ventilation, n (%)* | - | 0 (0)/4 (100) | 4 (100)/0 (0) |
| *Ventilation length (days)* | - | 8 [5-14] | 9 [5-11] |
| *Bilateral/extensive opacities (chest X-ray)* | - | 4 (100) | 4 (100) |
| *Acute organ dysfunction** | 0 (0) | 1 (25) | 2 (50) |
| *Erythropheresis* | 0 (0) | 2 (50) | 4 (100) |
| *Hospitalization length in ICU (days)* | 0 [0-0] | 9.5 [6-15] | 13 [10-32] |
| *Total hospitalization length (days)* | 7 [3-9] | 13 [9-19] | 20.5 [13-58] |
| *Hb level (g/dL)* | 9.3 [7.5-11] | 7.4 [6.1-7.8] | 8.6 [8.0-9.2] |
| *Reticulocyte count (G/L)* | 332 [217-365] | 387 [153-440] | 292 [214-465] |
| *Leukocyte count (G/L)* | 5.5 [5.2-11.3] | 16.3 [15.7-20.4] *^†^* | 24.0 [11.9-36.4] |
| *Absolute neutrophil count (G/L)* | 3.0 [1.6-4.8] | 10.4 [10.0-16.4] *^†^* | 19.2 [8.9-26.9] |
| *Monocyte count (G/L)* | 0.8 [0.5-1.1] *^†^* | 2.0 [1.6-2.4] *^†^* | 2.7 [1.1-7.3] |
| *Platelets (G/L)* | 201 [155-283] | 260 [145-395] | 159 [140-241] |
| *CRP (mg/L)* | 9 [7-11] *^†^* | 161 [152-197] | 188 [147-198] |
| *AST (U/L)* | 49 [36-52] | 64 [32-190] | 50 [35-1474] |
| *Unconjugated bilirubin (µmol/L)* | 20 [12-48] | 32 [20-102] | 25 [23-56] |
| *LDH (U/L)* | 500 [381-620] *^†^* | 641 [437-857] *^†^* | 841 [450-1828] |

Data are expressed as median [range], or percentage.

*^†^* 1missing data. *liver, kidney, cardiac or neurological dysfunction

ACS: acute chest syndrome. AST: aspartate aminotransferase. CRP: C-reactive protein. G6PD: Glucose-6-phosphate dehydrogenase. Hb: hemoglobin. ICU: intensive care unit. LDH: lactate dehydrogenase. MET: monthly exchange transfusion. SCD: sickle cell disease. VOC: vaso-occlusive crisis.

**SUPPLEMENTARY FIGURES**

**
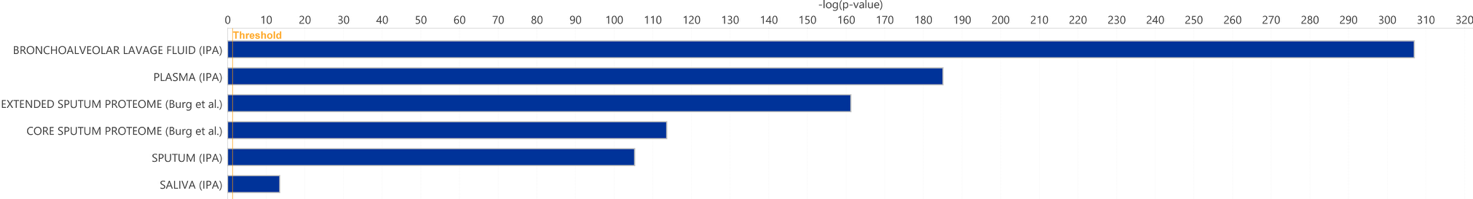
**

**Figure S1.** Quality assessment of samples performing Fisher exact test with terms related to biofluids from IPA and the 2 sputum proteome gold standards (Burg et al.).^3^ Icons from the Human Protein Atlas website (https://www.proteinatlas.org/).


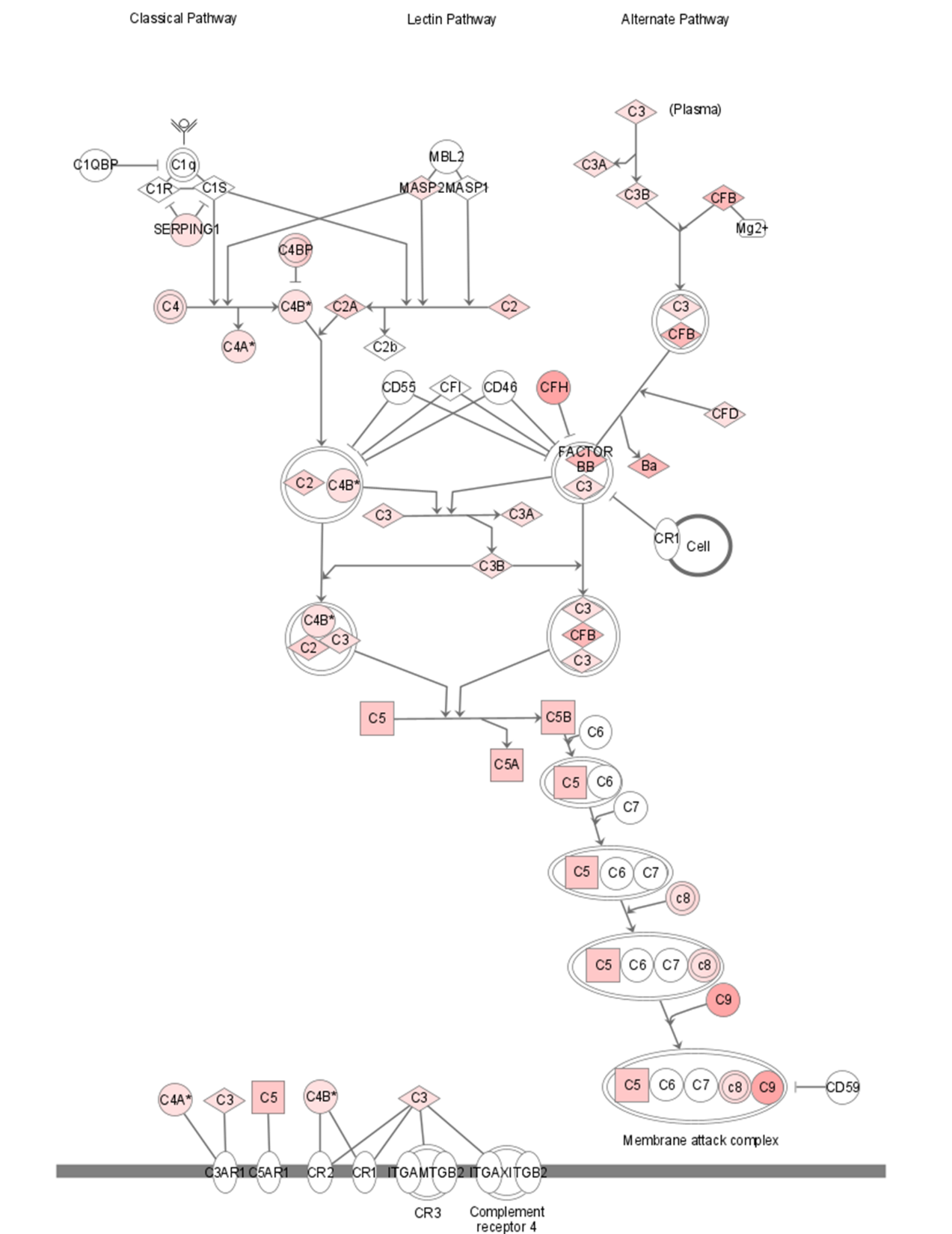


**Figure S2.** Ingenuity Pathways Analysis (IPA) of the complement system, in the proteomic analysis of sputum and tracheal aspirates from SCD patients during VOC (n=3), non-intubated ACS (n=4), and intubated ACS (n=4), overlayed with experimental expression values. Red nodes reflect up-regulation in ACS samples compared to VOC samples, whereas green nodes reflect down-regulation. The meaning of shapes and edges is described here: https://qiagen.my.salesforce-sites.com/KnowledgeBase/articles/Knowledge/Legend.


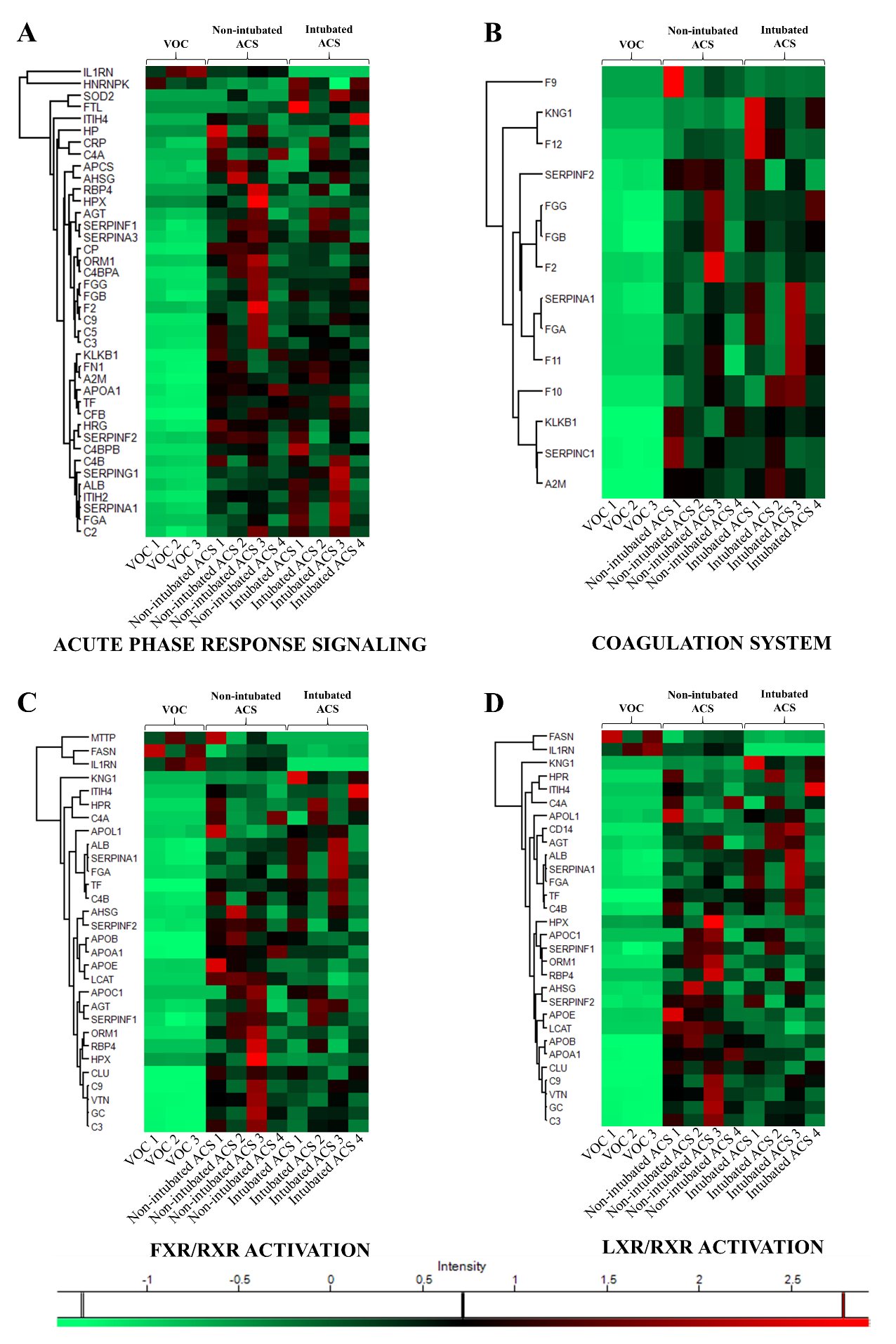


**Figure S3.** Heatmaps of proteins involved in the 4 other most enriched IPA pathways than the complement system, highlighted by proteomic analysis of sputum and tracheal aspirates from SCD patients during VOC (n=3), non-intubated ACS (n=4), and intubated ACS (n=4): Acute phase response signaling (A), Coagulation system (B), FXR/RXR activation (C), and LXR/RXR activation (D). Heatmaps were generated by Perseus. Hierarchical clustering was performed on z-scored Label-Free Quantification (LFQ) intensities using Pearson correlation with average linkage. ACS: acute chest syndrome. VOC: vaso-occlusive crisis.


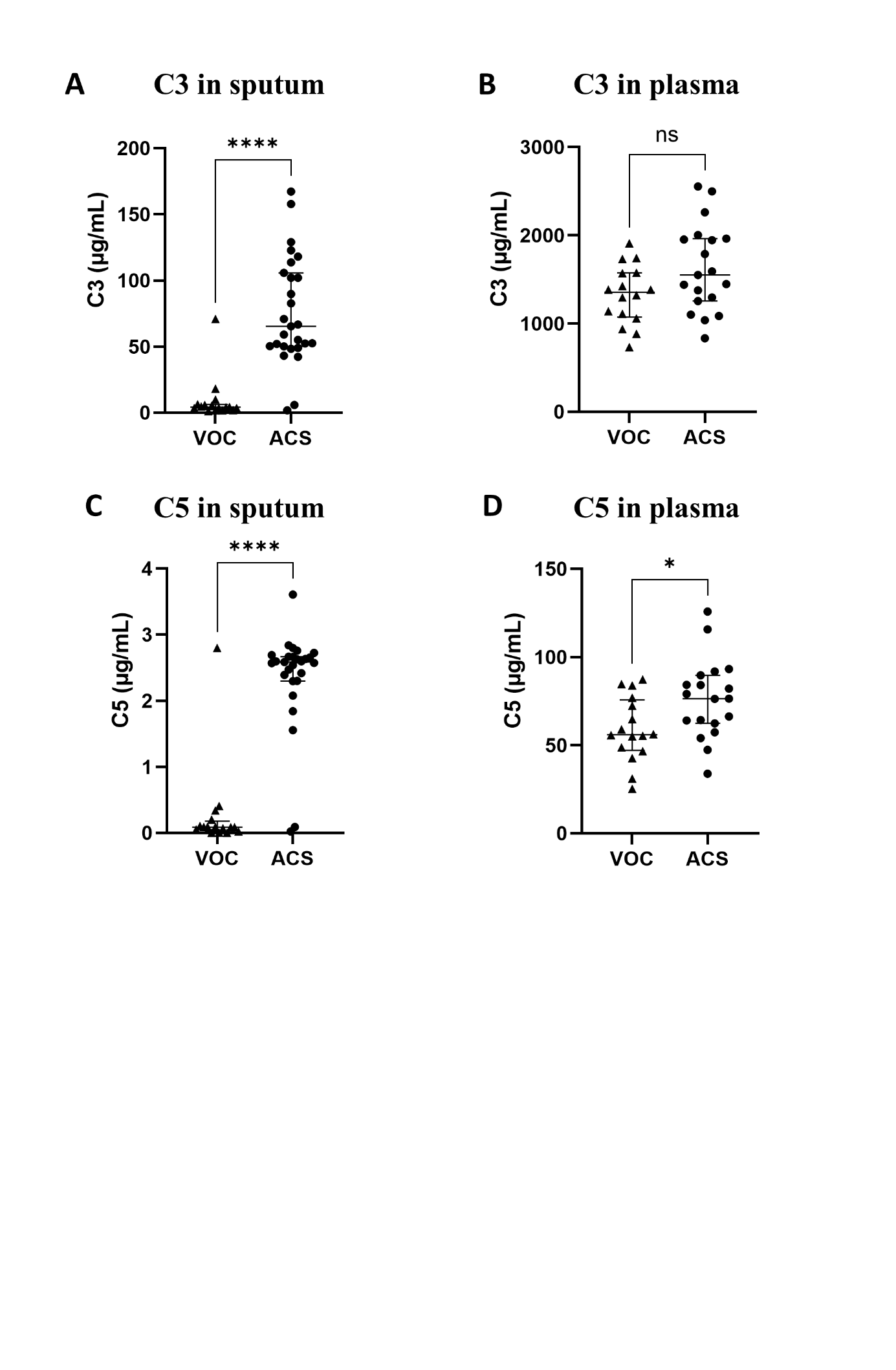
**Figure S4.** Comparison of the levels of C3 (A-B) and C5 (C-D) in sputum or tracheal aspirates (A, C) and in plasma (B, D) from SCD patients, between ACS (n= 27 episodes) and VOC without ACS (n=16 episodes). ACS: acute chest syndrome. VOC: vaso-occlusive crisis. *****p* < 0.0001; **p* < 0.05 by Mann-Whitney test.


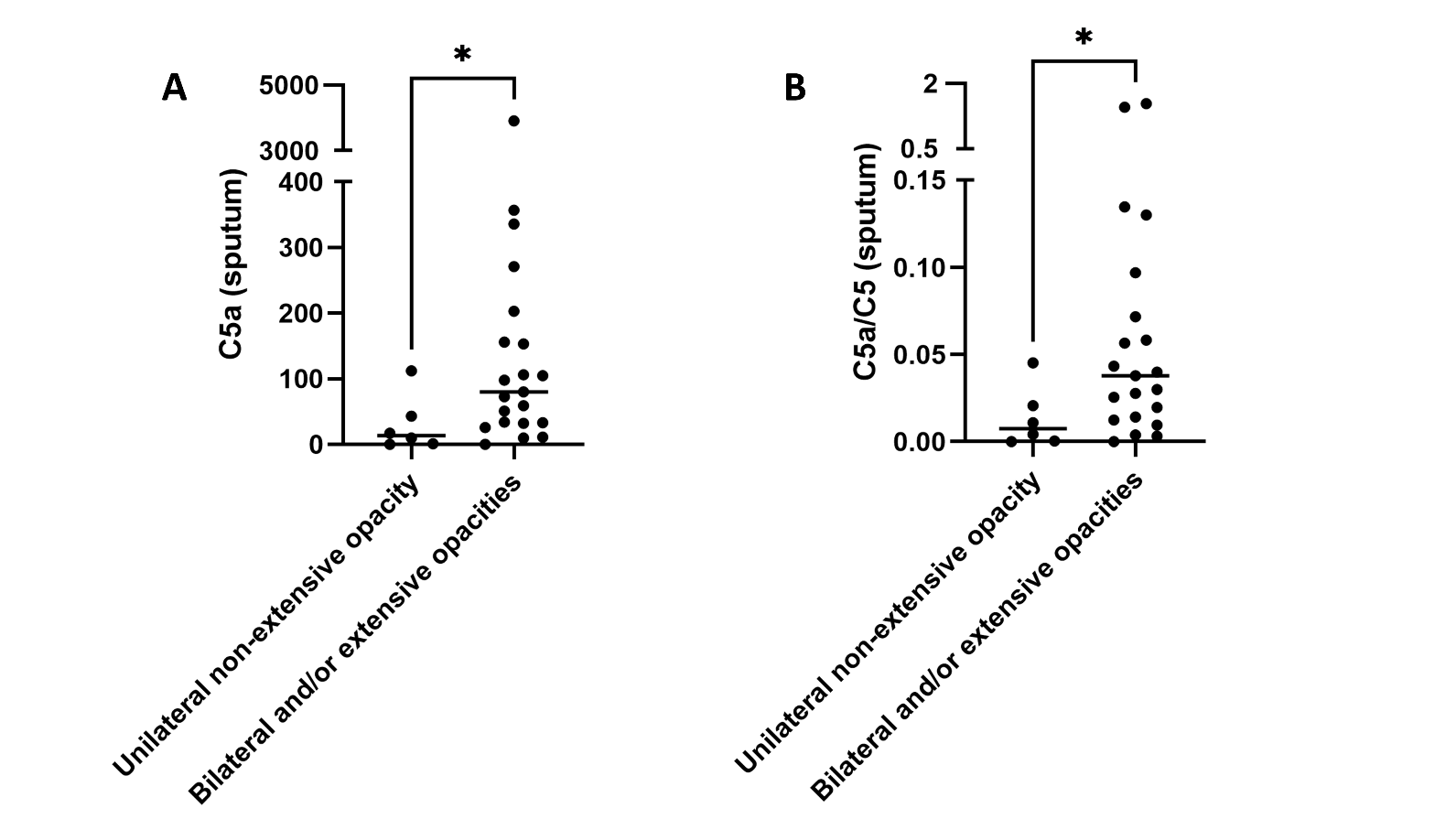


**Figure S5.** C5a (A) and C5a/C5 ratio (B) in sputum or tracheal aspirates from SCD patients during ACS (n= 27 episodes) with (n=21) or without (n=6) bilateral and/or extensive opacities. **p* < 0.05 by Mann-Whitney test.

**REFERENCES**

1. Cox J, Hein MY, Luber CA, Paron I, Nagaraj N, Mann M. Accurate proteome-wide label-free quantification by delayed normalization and maximal peptide ratio extraction, termed MaxLFQ. *Mol Cell Proteomics.* 2014;13(9):2513-2526.

2. Tyanova S, Temu T, Sinitcyn P, et al. The Perseus computational platform for comprehensive analysis of (prote)omics data. *Nat Methods.* 2016;13(9):731-740.

3. Burg D, Schofield JPR, Brandsma J, et al. Large-Scale Label-Free Quantitative Mapping of the Sputum Proteome. *J Proteome Res.* 2018;17(6):2072-2091.
